# Role of Electronic Correlations on Exchange Interactions and Curie Temperature in Monolayer CrI_3_

**DOI:** 10.1021/acsomega.6c02634

**Published:** 2026-05-28

**Authors:** Arthur Krindges, Carlos Alberto Vaz de Morais Junior, Maurício Jeomar Piotrowski

**Affiliations:** Department of Physics, 37902 Federal University of Pelotas, Pelotas, Rio Grande do Sul 96010-900, Brazil

## Abstract

We investigate the magnetic properties of monolayer CrI_3_ by combining first-principles density functional theory calculations with an effective spin model and finite-temperature statistical analysis. The electronic structure is computed within the DFT+*U* framework, including spin–orbit coupling, allowing us to assess the role of electronic correlations on the structural, electronic, and magnetic properties of the system. Magnetic exchange interactions are extracted using the Green’s-function-based Liechtenstein formalism and mapped onto an effective isotropic Heisenberg Hamiltonian. Our results show that the on-site Coulomb interaction *U* strongly influences the Cr–I bond geometry and the ligand-mediated superexchange pathways, leading to a nonmonotonic dependence of the dominant nearest-neighbor exchange interaction on *U*. This behavior places monolayer CrI_3_ in an intermediate-correlation regime, where electronic localization and hybridization compete to determine the strength of ferromagnetic coupling. Finite-temperature magnetic properties are investigated using the cluster mean-field theory, which partially incorporates short-range magnetic correlations beyond conventional mean-field approaches. The calculated effective Curie temperature within the cluster mean-field (CMF) framework exhibits a pronounced maximum at intermediate values of *U*, yielding values of the same order of magnitude as experimental measurements for monolayer CrI_3_. However, this temperature should be interpreted as an effective energy scale associated with the onset of short-range magnetic correlations within the CMF approximation, rather than a true thermodynamic transition temperature. These results indicate that the dominant energy scale governing magnetic ordering is primarily controlled by isotropic exchange interactions and short-range correlations. Thus, our results show that a minimal Heisenberg model parametrized from first principles, combined with the CMF theory, supplies a well-grounded and computationally efficient framework to describe the magnetic properties of two-dimensional van der Waals magnets.

## Graphical abstract

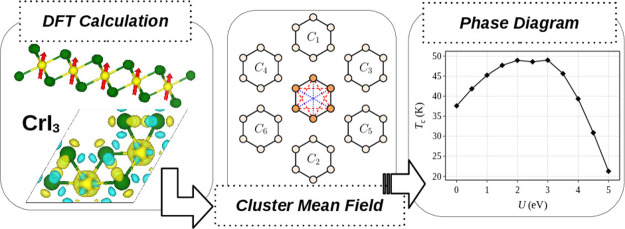

## Introduction

Spintronics has established itself as an important research field in condensed matter physics and materials science, aiming to exploit/control the electron spin degrees of freedom, at the microscopic level and in its collective manifestation (as magnetization), for the development of new functionalities and energy-efficient technologies.^1^ Therefore, a detailed microscopic understanding of spin-dependent electronic structure, magnetic exchange interactions, and finite-temperature behavior (thermal fluctuations) is fundamental to advancing the basic knowledge and the possible technological applications.

In this context, two-dimensional (2D) materials, particularly van der Waals (vdW) crystals, have emerged as promising platforms for possible spintronic applications.^2–4^ Unlike conventional ultrathin magnetic films, vdW crystals can be exfoliated down to the monolayer limit while preserving structural and electronic integrity. Their layered nature (weak interlayer bonding) enables the isolation of stable monolayers and the artificial heterostructure assembly with atomic-scale control. This versatility allows for the engineering of magnetic, electronic, and spin-transport properties via stacking, external fields, strain, or proximity effects. Furthermore, vdW materials can exhibit sizable magnetic anisotropy and strong relativistic effects (such as spin–orbit coupling), which play a decisive role in stabilizing long-range magnetic order in reduced dimensionality.^2,5^

Among the family of 2D magnetic vdW materials, chromium triiodide (CrI_3_) occupies a prominent position as one of the first systems in which intrinsic ferromagnetic order was experimentally demonstrated down to the monolayer limit.^6^ In this material, Cr^3+^ ions form a honeycomb lattice that constitutes the magnetic sublattice, while the magnetic ground state and interlayer coupling strongly depend on the thickness and stacking configuration.^7^ The coexistence of a layered crystal structure, sizable magnetic anisotropy, and tunability via external perturbations makes CrI_3_ an archetypal system for exploring low-dimensional magnetism and its potential applications in spintronic devices and vdW heterostructures.

Experimentally, monolayer CrI_3_ was first obtained by mechanical exfoliation from the bulk crystal, exhibiting a Curie temperature of approximately 45 K, compared to about 61 K in the three-dimensional phase.^6^ This reduction highlights the critical role of dimensionality and magnetic interactions in determining the thermal stability of the ferromagnetic phase. CrI_3_ is also a semiconductor with an experimental band gap of the order of 2.7 eV.^8^ From a theoretical perspective, however, a quantitative description of its electronic structure remains challenging within standard density functional theory (DFT). In particular, calculations based on the generalized gradient approximation (GGA), using the Perdew–Burke–Ernzerhof (PBE) functional, systematically underestimate the band gap, a limitation that persists even when on-site Hubbard corrections for the Cr d states and spin–orbit coupling (SOC) are included.^9,10^ More accurate treatments, such as hybrid functionals or beyond-DFT approaches, are computationally demanding and often impractical for large-scale or parameter-dependent studies.^11^

Despite these limitations, it is important to note that an accurate description of the conduction-band manifold is not a prerequisite for capturing the dominant magnetic interactions in CrI_3_. The exchange mechanisms governing its magnetism are primarily determined by the localized Cr 3d states near the Fermi level and by ligand-mediated superexchange pathways involving iodine atoms. Consequently, a DFT+*U* framework, when carefully analyzed and combined with an appropriate magnetic model, can provide a reliable and physically transparent description of the magnetic interactions and finite-temperature properties.

Some recent theoretical studies have provided important microscopic insights into the magnetic interactions in monolayer CrI_3_, among which we highlight the role of Cr–I–Cr superexchange pathways, orbital hybridization, and the applicability of Goodenough–Kanamori–Anderson (GKA) rules.^12–14^ Despite these advances, a unified framework that quantitatively connects electronic correlations, exchange interactions, and finite-temperature properties within a single computational scheme remains lacking. In particular, the quantitative relationship between the Hubbard parameter *U*, the resulting exchange couplings, and the Curie temperature has not yet been fully clarified.

In this work, we address this issue by investigating the monolayer CrI_3_ using first-principles calculations within the DFT+*U*+SOC approach. We systematically analyze the influence of the Hubbard parameter *U* on the electronic structure and structural properties of the system. Based on the converged electronic states, we extract magnetic exchange parameters using a Green’s-function-based formalism and map them onto an effective isotropic Heisenberg model. The resulting spin Hamiltonian is then solved using the cluster mean-field (CMF) method, which explicitly incorporates short-range magnetic correlations beyond the CMF theory, allowing for a reliable determination of thermodynamic quantities and an effective Curie temperature within the CMF framework.^15,16^

It is important to emphasize that DFT is employed as a physically grounded framework to derive material-specific microscopic parameters that cannot be fixed a priori within a purely phenomenological model. While effective spin Hamiltonians provide conceptual transparency, their predictive capability critically depends on the choice of input parameters, such as exchange interactions and local magnetic moments. In this context, the DFT+*U* approach plays a central role by explicitly linking electronic correlations to the underlying electronic structure and chemical bonding. By systematically varying the Hubbard parameter *U*, we are able to probe how the degree of localization of Cr 3d states, the Cr–I hybridization, and the associated superexchange pathways evolve, and how these changes propagate to the magnetic exchange interactions and, ultimately, to the effective Curie temperature.

Therefore, our combined first-principles and statistical mechanics framework provides a coherent microscopic-to-macroscopic description of magnetism in monolayer CrI_3_, elucidating the electronic correlation role in shaping exchange interactions and the finite-temperature magnetic order. The present approach offers a computationally efficient and physically transparent route for investigating magnetic phase transitions in 2D vdW magnets and can be readily extended to explore the effects of strain, doping, and external fields on related systems.

This paper is organized as follows. In the second section, we describe the computational details and the first-principles methodology employed to obtain the electronic structure and magnetic exchange parameters. We introduce the CMF approach used to solve the effective spin model. The results and their physical interpretation are presented in the third section, followed by the main conclusions in the fourth section.

## Computational Details and First-Principles Methods

Our first-principles calculations were performed, considering the DFT^17,18^ framework, to investigate the electronic, structural, and magnetic properties of the monolayer CrI_3_. All calculations were carried out using the *OpenMX* code,^19^ which employs a localized pseudoatomic orbital basis and allows for an efficient and accurate treatment of magnetic systems with strong electronic correlations.

Exchange–correlation effects were described within the GGA-PBE functional.^20^ Local electronic correlations associated with the Cr 3d states were taken into account through the DFT+*U* approach,^21,22^ with the Hubbard parameter *U* systematically varied in the range from 0 to 5 eV. SOC was explicitly included in all calculations, as relativistic effects are known to play an important role in the magnetic properties of CrI_3_. Long-range van der Waals interactions were incorporated using Grimme’s semiempirical D3 correction scheme.^23,24^ Although van der Waals corrections are primarily relevant for interlayer interactions, their inclusion at the D3 level leads only to minor quantitative changes in the intralayer structural parameters of the monolayer (below 1%), without affecting the overall trends or the physical conclusions. The computational overhead associated with the D3 correction is negligible, and its inclusion ensures a consistent treatment of dispersive interactions.

The Brillouin zone was sampled using a 10 × 10 × 1 Monkhorst–Pack **k**-point mesh. The real-space grid cutoff was set to 400 Ry, and the total energy convergence criterion was fixed at 10^–9^ Hartree. Structural relaxations were performed until the residual forces on each atom were smaller than 10^–4^ Hartree/Bohr. To avoid spurious interactions between periodic images along the out-of-plane direction, a vacuum spacing of 21 Å was introduced. These computational parameters ensure well-converged structural, electronic, and magnetic properties across the entire range of Hubbard *U* values considered.

Figure 1 illustrates the optimized atomic structure and unit cell of the CrI_3_ monolayer. The Cr atoms form a honeycomb lattice, which forms the magnetic sublattice of the system, while the I atoms mediate the dominant superexchange interactions between neighboring Cr sites.

**Figure 1:**
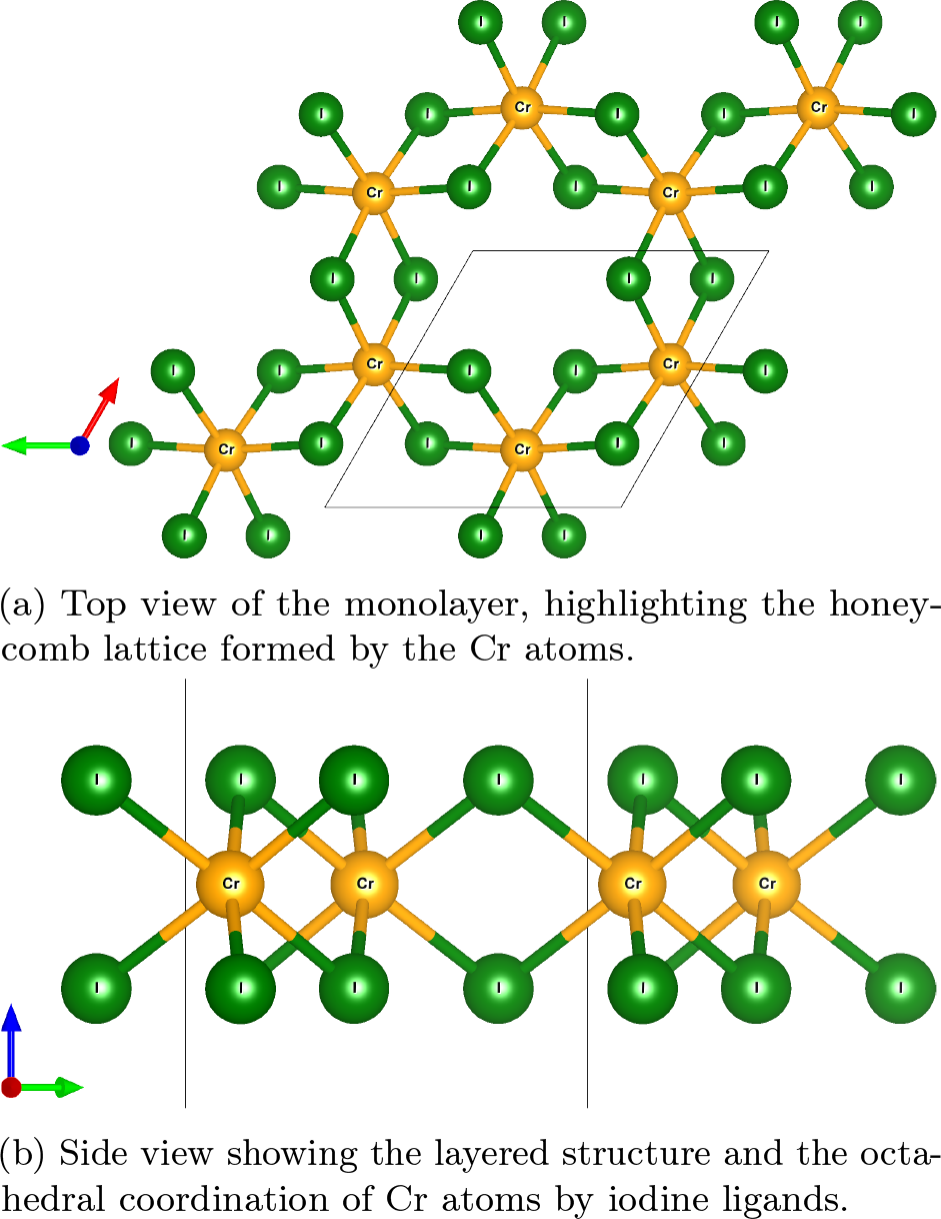
Optimized atomic structure of the CrI_3_ monolayer. (a) In-plane view, where Cr atoms form a honeycomb lattice that constitutes the magnetic sublattice of the system. (b) Out-of-plane view illustrating the layered geometry and the CrI_6_ octahedra that mediate superexchange interactions between neighboring Cr sites.

### Extraction of Magnetic Exchange Interactions

The magnetic exchange parameters were obtained from the converged DFT+*U*+SOC electronic structures using the *TB2J* package.^25^ This approach maps the *ab initio* electronic Hamiltonian onto an effective Heisenberg model by evaluating the magnetic response of the system within a Green’s-function formalism. For this purpose, the electronic states around the Fermi level are represented in a localized basis, enabling the construction of an effective tight-binding Hamiltonian suitable for describing magnetic interactions.

The isotropic exchange interactions *J*_*ij*_ were computed using the Liechtenstein–Katsnelson–Antropov–Gubanov (LKAG) formalism,^26^ in which the exchange parameters correspond to the second-order variation of the total energy with respect to infinitesimal rotations of the local magnetic moments. Within this framework, the exchange interaction between sites *i* and *j* is given by

$$J_{ij}=\frac{1}{\pi }Im\;\int_{-\infty }^{E_{F}}\mathrm{Tr}[\Delta_{i}G_{ij}^{↑}(E)\Delta_{j}G_{ji}^{↓}(E)]dE$$ 1

where *E*_F_ is the Fermi energy, *G*_*ij*_^σ^(*E*) denotes the real-space Green’s function for spin σ, and Δ_*i*_ represents the local exchange splitting at site *i*. The resulting exchange constants provide a direct and physically transparent connection between the underlying electronic structure and the effective spin model.

## Cluster Mean-Field Approach

To investigate the finite-temperature magnetic and thermodynamic properties of monolayer CrI_3_, we considered an effective Heisenberg model with spin *S* = 1/2 on the honeycomb lattice, including first- (*J*_1_), second- (*J*_2_), and third-nearest-neighbor (*J*_3_) exchange interactions.^27,28^ Previous studies and our *ab initio* results indicate that interactions beyond third neighbors play a negligible role in the magnetic behavior of the system.^29^ The Hamiltonian is written as follows:

$$H=-J_{1}\sum_{\langle ij\rangle_{1}}{\mathrm{S⃗}}_{i}\cdot {\mathrm{S⃗}}_{j}-J_{2}\sum_{\langle ij\rangle_{2}}{\mathrm{S⃗}}_{i}\cdot {\mathrm{S⃗}}_{j}-J_{3}\sum_{\langle ij\rangle_{3}}{\mathrm{S⃗}}_{i}\cdot {\mathrm{S⃗}}_{j}$$ 2

Furthermore, the choice of *S* = 1/2 corresponds to an effective spin normalization, with the magnitude of the local moment absorbed into the exchange parameters.

Although the physical Cr^3+^ ions correspond to a high-spin configuration with *S* = 3/2, the present choice of *S* = 1/2 should be understood as an effective normalization of the spin degrees of freedom. In this framework, the magnitude of the local magnetic moment is absorbed into the exchange parameters, which are obtained from first-principles calculations, assuming a classical spin normalization. As a result, the relevant magnetic energy scales entering the thermodynamic description are preserved, ensuring consistency between the *ab initio* parametrization and the statistical model.

Since the above Hamiltonian does not admit an exact analytical solution, we employed the CMF approach to capture thermal magnetic properties beyond the CMF theory. In this method, the lattice is partitioned into finite clusters, each treated exactly, while the interactions between different clusters are approximated at the mean-field level. The system is solved exactly within an isolated cluster containing a finite number of interactions, described by $H_{C}$ , while the interactions between clusters are treated through the mean-field approximation ${\mathrm{S⃗}}_{i}\cdot {\mathrm{S⃗}}_{j}\approx {\mathrm{S⃗}}_{i}\cdot {\mathrm{m⃗}}_{j}+{\mathrm{m⃗}}_{i}\cdot {\mathrm{S⃗}}_{j}-{\mathrm{m⃗}}_{i}{\mathrm{m⃗}}_{j}$ , represented by $H_{\mathrm{MF}}$ .^30^ Thus, the effective Hamiltonian in the CMF formalism can be written as follows:

$$H_{\mathrm{CMF}}=H_{C}+H_{\mathrm{MF}}$$ 3

$$H_{C}=-J_{1}\sum_{\langle ij\rangle_{1}}^{n}{\mathrm{S⃗}}_{i}\cdot {\mathrm{S⃗}}_{j}-J_{2}\sum_{\langle ij\rangle_{2}}^{n}{\mathrm{S⃗}}_{i}\cdot {\mathrm{S⃗}}_{j}-J_{3}\sum_{\langle ij\rangle_{3}}^{n}{\mathrm{S⃗}}_{i}\cdot {\mathrm{S⃗}}_{j}$$ 4

$$H_{\mathrm{MF}}=-J_{1}\sum_{\langle ij^{\prime }\rangle_{1}}^{n}{\mathrm{S⃗}}_{i}^{\prime }\cdot {\mathrm{m⃗}}_{j^{\prime }}-J_{2}\sum_{\langle ij^{\prime }\rangle_{2}}^{n}{\mathrm{S⃗}}_{i}^{\prime }\cdot {\mathrm{m⃗}}_{j^{\prime }}-J_{3}\sum_{\langle ij^{\prime }\rangle_{3}}^{n}{\mathrm{S⃗}}_{i}^{\prime }\cdot {\mathrm{m⃗}}_{j^{\prime }}$$ 5

with ${\mathrm{S⃗}}_{i}^{\prime }={\mathrm{S⃗}}_{i}-\frac{1}{2}{\mathrm{m⃗}}_{i}$ , *n* = 6 and *j*′ representing the interaction with the element *j* of a neighboring cluster.

The effective CMF Hamiltonian can be expressed as follows:

$$H_{\mathrm{CMF}}=H_{C}+H_{\mathrm{MF}}$$ 6

where $H_{C}$ contains all exchange interactions within a cluster of *n* = 6 spins, and $H_{\mathrm{MF}}$ accounts for the coupling between the cluster and its neighbors through self-consistently determined mean fields. Figure 2 schematically illustrates the CMF construction, where the central cluster interacts with replicas of itself via effective boundary fields. By explicitly incorporating short-range magnetic correlations and lattice geometry, the CMF approach provides a substantial improvement over the standard mean-field theory, which is particularly important for the quantitative description of magnetic ordering in low-dimensional systems, such as monolayer CrI_3_.

**Figure 2:**
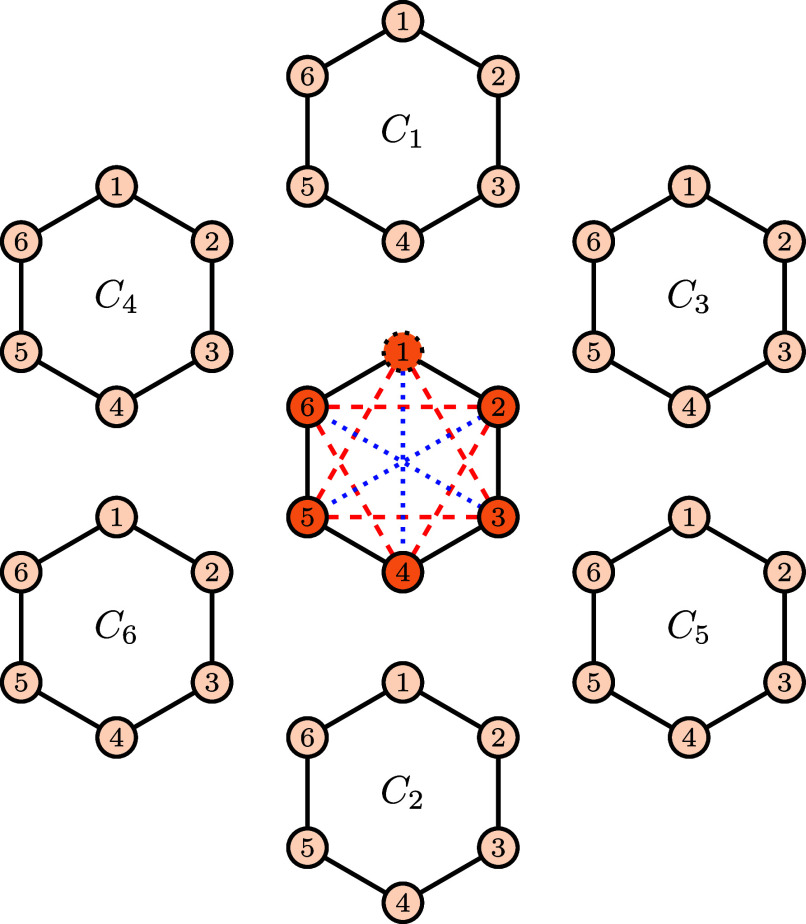
Schematic representation of the honeycomb lattice within the CMF formalism. The central cluster consists of *n* = 6 Cr spins and is treated exactly, explicitly including first- (*J*_1_), second- (*J*_2_), and third-nearest-neighbor (*J*_3_) interactions, depicted by black, red, and blue bonds, respectively. Interactions with spins belonging to neighboring clusters *C*_*i*_ are incorporated through self-consistent mean fields, represented by replicas of the central cluster.

The choice of a cluster with *n* = 6 sites corresponds to the minimal unit that preserves the honeycomb lattice geometry and explicitly includes exchange interactions up to the third neighbor. Since the magnetic interactions in monolayer CrI_3_ are dominated by short-range couplings, the CMF results are expected to converge rapidly with an increasing cluster size. Therefore, while larger clusters may lead to quantitative refinements, the qualitative behavior of thermodynamic quantities, including the position of the Curie temperature maximum, is not expected to be significantly affected.

## Results and Discussion

### Structural and Electronic Properties

The optimized crystal structure of monolayer CrI_3_ was obtained within the DFT+*U* framework, including spin–orbit coupling. For all investigated values of the on-site Coulomb interaction *U*, the ground state converges to a ferromagnetic configuration, with magnetic moments predominantly localized on the Cr atoms, in agreement with experimental observations and previous theoretical reports.^9^

Figure 1 displays the relaxed atomic structure of the monolayer, while Figure 3 presents the evolution of the main structural parameters as a function of *U*. A clear and systematic expansion of the Cr–I bond length is observed with increasing *U*, accompanied by a gradual reduction of the Cr–I–Cr bond angle. This trend is a direct consequence of the enhanced localization of the Cr 3d electrons induced by stronger on-site Coulomb repulsion, which weakens the Cr–I covalent hybridization and modifies the superexchange pathways mediating magnetic interactions.

**Figure 3:**
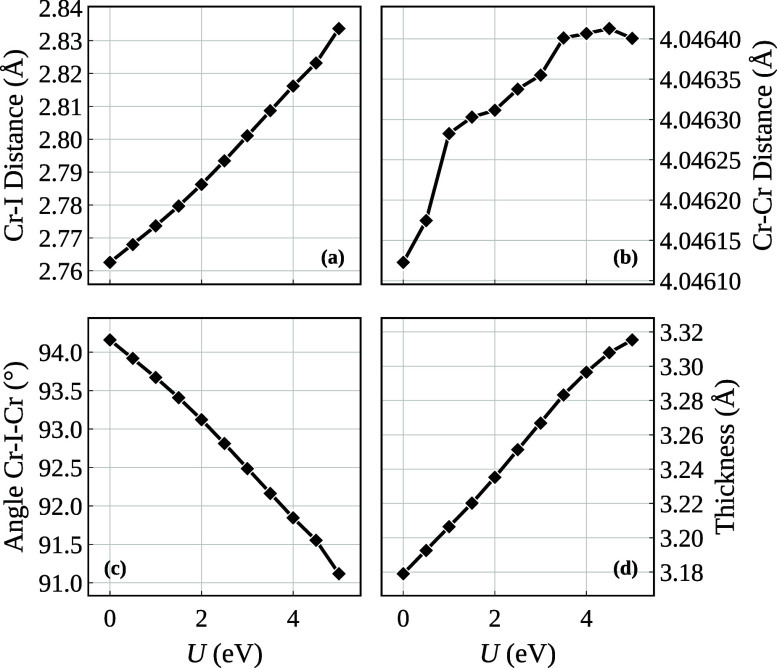
Evolution of the structural parameters of the CrI_3_ monolayer as a function of the Hubbard parameter *U*, obtained from fully relaxed DFT+*U*+SOC calculations. (a) Cr–I bond length, (b) in-plane Cr–Cr distance, (c) Cr–I–Cr bond angle, and (d) monolayer thickness.

The evolution of the electronic states with increasing Hubbard *U* can be inferred from the calculated energy gap and local magnetic moments presented in Figure 4. The valence states near the Fermi level remain dominated by strongly hybridized Cr 3d and I 5p orbitals across the entire *U* range, indicating that the fundamental electronic character of monolayer CrI_3_ is preserved. This robust hybridization confirms that the dominant magnetic exchange mechanism is governed by ligand-mediated Cr–I–Cr superexchange rather than by direct Cr–Cr interactions. Consequently, despite known limitations of the GGA+*U* framework in describing unoccupied states, the occupied electronic manifold relevant for magnetic interactions is reliably captured, ensuring the physical consistency of the extracted exchange parameters.

**Figure 4:**
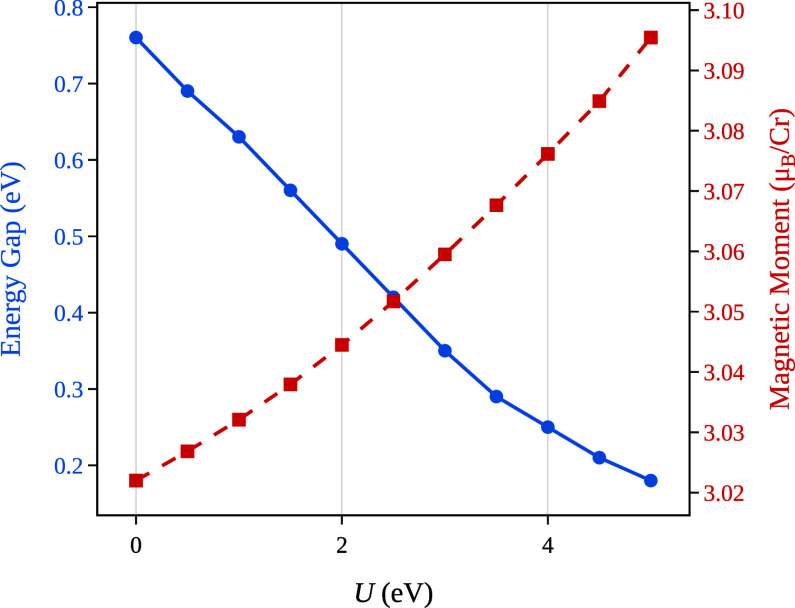
Evolution of the fundamental band gap and the local magnetic moment per Cr atom as a function of the Hubbard parameter *U*, obtained within the DFT+*U*+SOC framework. The energy gap corresponds to the spin-polarized gap at the Fermi level, while the magnetic moment is evaluated within the Cr atomic spheres.

From a chemical perspective, the magnetic interactions in monolayer CrI_3_ can be understood in terms of ligand-mediated superexchange mechanisms involving Cr 3d and I 5p orbitals. The strength and sign of the exchange coupling are directly linked to the degree of covalent hybridization along the Cr–I–Cr bonding network and to the corresponding bond geometry. In particular, the Cr–I–Cr bond angle plays a key role in determining the efficiency of orbital overlap, in accordance with the GKA rules,^31–33^ as discussed in recent studies of monolayer CrI_3_.^12–14^ As the Hubbard *U* parameter increases, the progressive localization of the Cr 3d electrons reduces the covalent character of the Cr–I bonds, modifying the orbital hybridization and, consequently, the superexchange pathways. This provides a chemically intuitive interpretation of how electronic correlations influence magnetic interactions in this system.

For *U* = 0, the calculated gap is approximately 0.8 eV, significantly smaller than the experimental value, yet consistent with previous GGA-PBE-based studies. The magnetic moment per Cr atom is close to 3 μ_B_, in agreement with the expected Cr^3+^ (3d^3^) electronic configuration and with values reported in the literature. As *U* increases, the magnetic moment exhibits a weak enhancement, reaching values close to 3.1 μ_B_, reflecting the increased localization of the Cr 3d states. In contrast, the insulating gap shows a gradual reduction, reaching approximately 0.2 eV for *U* = 5 eV. Although counterintuitive within a simple atomic picture, this behavior originates from the relative shift of the conduction bands and the strong hybridization between Cr 3d and I 5p states, which is not fully corrected within the GGA+*U* framework. This behavior highlights the limitations of a static GGA+*U* correction in describing the unoccupied states of CrI_3_ and should not be interpreted as a literal reduction of the true quasiparticle gap.

Importantly, the underestimation of the band gap does not compromise the reliability of the extracted magnetic exchange parameters. The dominant exchange interactions are primarily determined by the occupied states near the Fermi level and by the degree of local magnetic polarization on the Cr sites. While more advanced approaches, such as hybrid functionals, are required for an accurate description of the conduction bands, the valence-band manifold and the Cr–I–Cr superexchange pathways are already well captured within the present DFT+*U*+SOC framework. As a result, the determination of effective magnetic interactions remains qualitatively and quantitatively reliable.

### Exchange Interactions from *Ab Initio* Calculations

The magnetic exchange interactions were evaluated using the Green’s-function-based Liechtenstein formalism as implemented in the *TB2J* package, starting from the converged DFT+*U*+SOC electronic structure. The resulting exchange constants *J*_*ij*_ were mapped onto an effective Heisenberg Hamiltonian

$$H=-\sum_{i\neq j}J_{ij}S_{i}\cdot S_{j}$$ 7

where **S**_*i*_ denotes a spin operator.

To provide a more explicit microscopic interpretation of the exchange interactions obtained from *TB2J*, it is instructive to analyze their origin in terms of the underlying electronic structure and chemical bonding. In monolayer CrI_3_, the dominant magnetic coupling arises from ligand-mediated superexchange pathways involving Cr 3*d* and I 5*p* orbitals. The strength and sign of these interactions are governed by the degree of Cr–I hybridization and by the Cr–I–Cr bond geometry, in accordance with the GKA rules.

Within this framework, the Hubbard parameter *U* plays a dual role. On one hand, increasing *U* enhances the localization of Cr 3*d* states and strengthens the local magnetic moments, which tends to reinforce ferromagnetic superexchange at intermediate coupling. On the other hand, larger *U* reduces the covalent character of the Cr–I bonds, weakening orbital hybridization and thus suppressing the efficiency of the superexchange mechanism. The resulting competition between these two effects provides a microscopic explanation for the nonmonotonic dependence of the nearest-neighbor exchange interaction *J*_1_ on *U*. In this sense, the TB2J-derived exchange parameters do not merely reproduce known trends, but allow us to quantitatively connect electronic correlations, chemical bonding, and magnetic interactions within a unified first-principles framework.

Since *TB2J* provides exchange parameters assuming a classical normalization |**S**| = 1, whereas the present model adopts a quantum mechanical description with *S* = 1/2, the exchange constants were rescaled accordingly to ensure consistency between the *ab initio* parametrization and the statistical treatment employed in the thermodynamic analysis.

The extraction of magnetic exchange interactions from the first-principles calculations involves projecting the electronic Hamiltonian onto a localized basis (e.g., Wannier functions, pseudoatomic orbitals, or projector-based methods), which may lead to quantitative differences in the absolute values of the exchange parameters. However, the overall trends as a function of the Hubbard *U* parameter are expected to be robust, as they are governed by fundamental physical mechanisms, that is, they are intrinsic to the electronic structure and chemical bonding. Consequently, the qualitative trends reported here are not expected to be dependent on the specific projection scheme employed.

Figure 5 shows the dependence of the first-, second-, and third-nearest-neighbor exchange interactions (*J*_1_, *J*_2_, and *J*_3_) on the Hubbard parameter *U*. For all values considered, the nearest-neighbor interaction *J*_1_ remains ferromagnetic and clearly dominates the magnetic energy scale, while *J*_2_ and *J*_3_ provide smaller but non-negligible contributions. The sign reversal observed for the third-nearest-neighbor exchange interaction suggests a competition between different superexchange channels, which may involve distinct orbital contributions. A detailed orbital-resolved decomposition of the exchange interactions, for instance, within the *TB2J* framework, would be highly valuable to further elucidate the microscopic origin of this behavior and will be addressed in future work.

**Figure 5:**
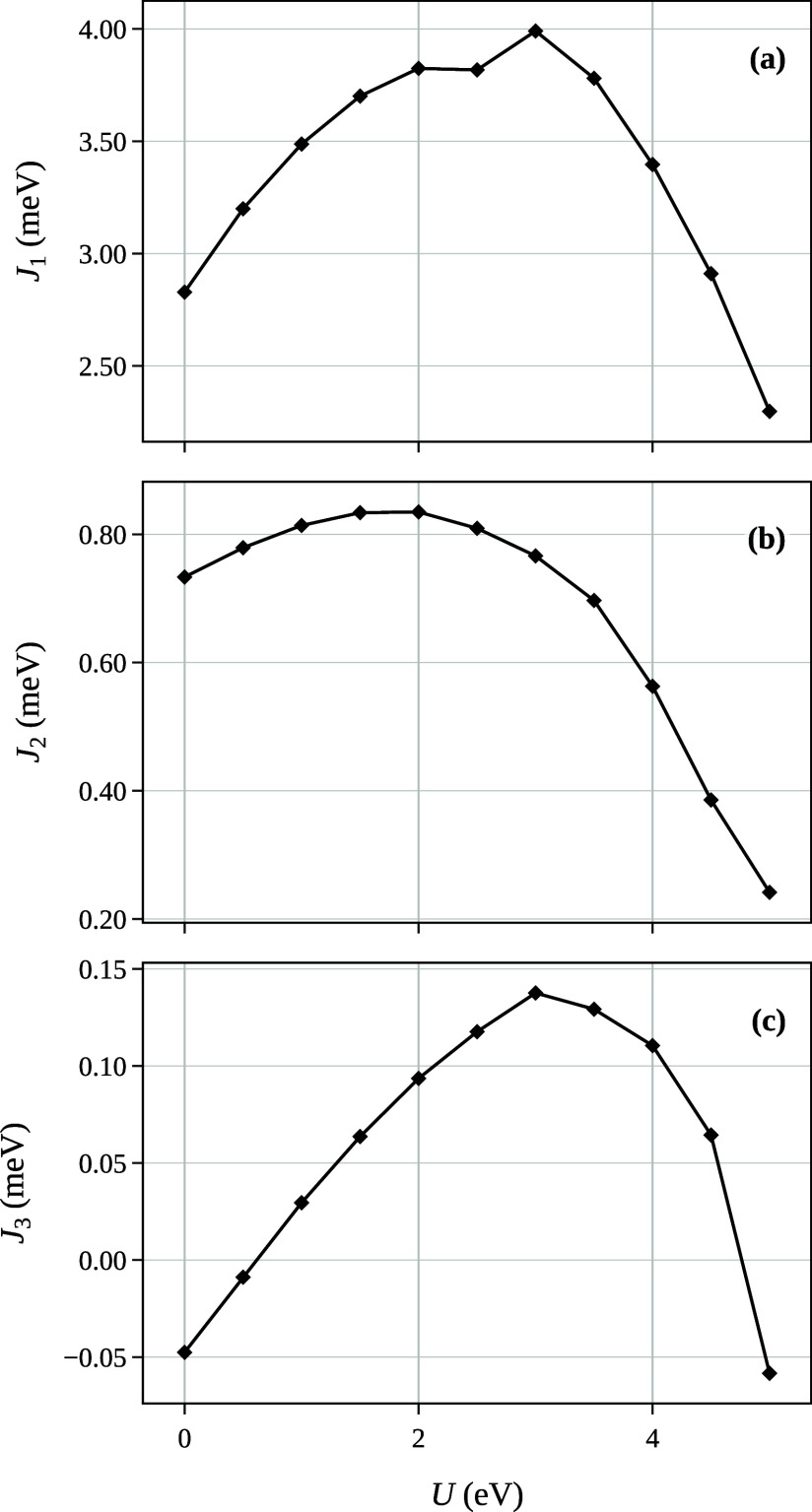
Hubbard-*U* dependence of the isotropic magnetic exchange interactions in monolayer CrI_3_, obtained from DFT+*U*+SOC calculations using the Liechtenstein formalism as implemented in the *TB2J* package. Panels (a), (b), and (c) show the first- (*J*_1_), second- (*J*_2_), and third-nearest-neighbor (*J*_3_) exchange parameters, respectively. Positive values of *J* correspond to ferromagnetic coupling.

A nonmonotonic dependence of *J*_1_ on *U* is observed. In the weak-coupling regime, increasing *U* enhances the local magnetic moments and stabilizes ferromagnetic superexchange, leading to an increase of *J*_1_. Beyond an intermediate coupling regime, however, *J*_1_ decreases as *U* becomes large, consistent with the expected *t* ^2^/*U* behavior of superexchange interactions in the strong-coupling limit. This places monolayer CrI_3_ in an intermediate-correlation regime, where electronic localization and ligand-mediated hybridization compete in determining the strength of magnetic interactions. The inclusion of SOC in the underlying electronic structure is essential for an accurate description of magnetic anisotropy, but its impact on the isotropic exchange constants is found to be secondary. As a result, the low-energy magnetic properties relevant for the thermal stability of the ferromagnetic phase can be reliably captured by the isotropic Heisenberg model considered here.

Moreover, the exchange parameters obtained for the monolayer are of the same order of magnitude as those reported for bulk CrI_3_.^29^ This similarity is consistent with the relatively close experimental Curie temperatures of the two systems, approximately 45 K for the monolayer and 60 K for the three-dimensional crystal.^6,34^ Such an agreement reinforces the robustness of the extracted exchange interactions and indicates that the dominant magnetic couplings are largely preserved upon dimensional reduction.

### Curie Temperature and Thermodynamic Properties from Cluster Mean-Field Theory

The rescaled exchange parameters were subsequently employed within a CMF approach to investigate the finite-temperature magnetic properties. This method partially incorporates short-range magnetic correlations beyond the CMF theory, which are essential for an adequate description of 2D magnetic systems.

It is important to clarify the physical interpretation of the Curie temperature obtained within the present isotropic Heisenberg model. In strictly 2D systems, the Mermin–Wagner theorem^35^ establishes that a long-range magnetic order at a finite temperature cannot occur in the absence of magnetic anisotropy. Therefore, the present model is not intended to describe the mechanism responsible for stabilizing the long-range order in monolayer CrI_3_, but rather to capture the dominant magnetic energy scale associated with isotropic exchange interactions.

Therefore, the effective Curie temperature (*T*_c_) extracted from the CMF calculations should be interpreted as an effective temperature scale governing the development of short-range magnetic correlations. In real materials, weak magnetic anisotropy, originating from SOC, acts as a symmetry-breaking perturbation that enables long-range order, while the overall magnitude of the ordering temperature is primarily determined by the strength of the isotropic exchange interactions. As a result, the present approach provides a reliable estimate of the characteristic energy scale of magnetic ordering even though anisotropic terms are not explicitly included in the model.

Figure 6 presents the temperature dependence of the normalized magnetization and the entropy per cluster for representative values of the Hubbard parameter: *U* = 0 and *U* = 3 eV. At zero temperature, the magnetization is fully saturated, assuming its maximum value |*m*|_s_, and decreases continuously with increasing temperature until it vanishes at *T*_c_, signaling the transition to the paramagnetic phase.

**Figure 6:**
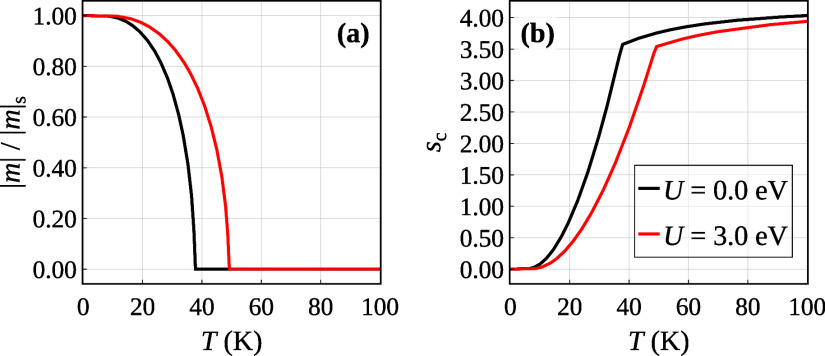
Temperature dependence of the normalized magnetization |*m*|/|*m*|_s_ (a) and the entropy per cluster *S*_c_ (b), obtained within the CMF approach for *U* = 0 eV (black) and *U* = 3 eV (red).

The entropy exhibits a complementary behavior, remaining nearly constant at low temperatures and showing a pronounced increase as the system approaches *T*_*c*_, followed by a smooth monotonic growth in the high-temperature paramagnetic regime. While the qualitative behavior of both observables remains unchanged with an increase in *U*, a clear shift of the transition temperature is observed. In particular, *T*_*c*_ increases from approximately 38 K for *U* = 0 eV to about 50 K for *U* = 3 eV, reflecting the effective strengthening of the exchange interactions in the intermediate correlation regime.

Figure 7 summarizes the calculated effective Curie temperature as a function of the Hubbard parameter *U.* The resulting *T*_c_ exhibits a pronounced maximum at intermediate values of *U*, closely following the behavior of the dominant nearest-neighbor exchange interaction *J*_1_. For *U* values in the range of 2–3 eV, the calculated *T*_c_ values are comparable to experimental measurements for monolayer CrI_3_, which report *T*_c_ ≈ 45 K,^3,6^ although such agreement should not be interpreted as a direct quantitative prediction, given the absence of long-wavelength fluctuations in the CMF approximation.

**Figure 7:**
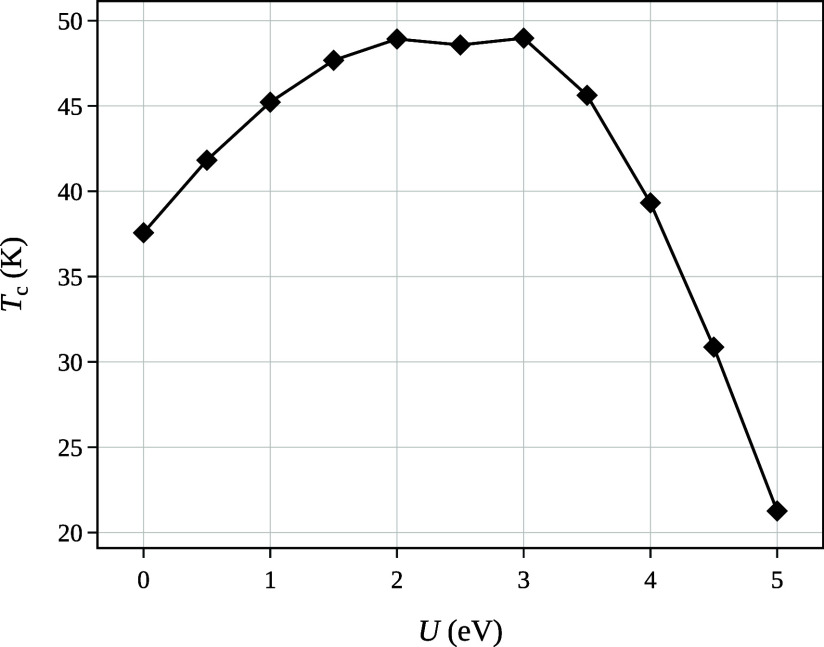
Effective Curie temperature, *T*_c_ (CMF), of monolayer CrI_3_ as a function of the Hubbard parameter *U*, obtained from CMF calculations based on exchange parameters derived from DFT+*U*+SOC.

We stress that the agreement between calculated and experimental *T*_c_ values should not be interpreted as a “strict quantitative prediction”, but rather as an indication that the dominant magnetic energy scales are correctly captured in the present framework. The approach used here involves a number of approximations, e.g., the use of the DFT+*U* method to describe the electronic correlations, the neglect of explicit magnetic anisotropy terms, and the use of the CMF approximation to describe finite-temperature properties. These approximations affect different aspects of the problem and do not necessarily introduce systematic errors in the same direction. Therefore, the consistency of the *T*_c_ obtained for intermediate values of *U*, together with the physically meaningful trends observed across the entire *U* range, suggests that the dominant magnetic energy scales are reasonably captured within the present framework, although the quantitative agreement should be interpreted with caution due to the limitations of the CMF approximation.

We note that the quantitative *T*_c_ value may depend on both the effective spin normalization and the cluster size employed in the CMF approach. However, these factors primarily affect the absolute scale of *T*_c_, while the overall trends as a function of the Hubbard parameter *U*, including the existence and position of the maximum, remain robust. This supports the interpretation that the observed behavior is governed by the underlying evolution of the exchange interactions rather than specific modeling choices.

The decrease of *T*_c_ at larger *U* can be directly traced back to the reduction of the exchange interactions due to excessive electronic localization, which weakens the superexchange mechanism. Conversely, at small *U*, the underestimation of correlation effects leads to reduced magnetic moments and less robust ferromagnetic coupling. These results point out the delicate balance between electronic correlations and ligand-mediated hybridization that governs the magnetic ordering temperature in monolayer CrI_3_.

Importantly, this agreement is obtained within an isotropic framework, indicating that isotropic exchange interactions set the dominant energy scale, while anisotropy remains essential for stabilizing the true long-range order in two dimensions. These results demonstrate that a minimal Heisenberg model, parametrized from first-principles calculations and treated within the CMF theory, is able to provide a reliable low-energy description of the magnetic properties of this 2D van der Waals magnet.

## Conclusions

In this work, we have presented a comprehensive investigation of the magnetic properties of monolayer CrI_3_ by combining first-principles electronic structure calculations with an effective spin model and finite-temperature statistical analysis. Using the DFT+*U* approach, including SOC, we analyzed how electronic correlations affect the structural, electronic, and magnetic characteristics of the system, with particular emphasis on the extraction of magnetic exchange interactions. Our results show that the on-site Coulomb interaction *U* plays a central role in controlling the degree of localization of the Cr 3d electrons, which in turn modifies the Cr–I bond geometry and the associated superexchange pathways. The dominant nearest-neighbor exchange interaction exhibits a nonmonotonic dependence on *U*, reflecting the competition between enhanced local magnetic moments at intermediate coupling and the reduction of superexchange strength in the strong-coupling limit. This behavior places monolayer CrI_3_ in an intermediate-correlation regime, consistent with its mixed ionic–covalent bonding character. By employing the CMF theory, we investigated the finite-temperature magnetic properties and obtained effective Curie temperatures (within CMF) that are comparable in magnitude to experimental values for intermediate values of *U*, representing the dominant isotropic exchange energy scale rather than a true thermodynamic transition temperature. The calculated temperature dependence of the magnetization and entropy captures the expected behavior across the ferromagnetic–paramagnetic transition, giving prominence to the short-range magnetic correlations in 2D systems. It is noteworthy that this agreement is achieved within an isotropic Heisenberg model, without requiring the inclusion of anisotropic exchange or Kitaev-type interactions. While magnetic anisotropy is essential for stabilizing the long-range order in strictly 2D systems, the present analysis focuses on the dominant isotropic exchange interactions that control the overall energy scale of the effective Curie temperature. In general, our results demonstrate that a minimal Heisenberg Hamiltonian parametrized from first-principles calculations, when combined with the CMF theory, provides a trustworthy and physically transparent low-energy description of the magnetic properties of monolayer CrI_3_. This framework offers a computationally efficient route to investigate magnetism in related 2D van der Waals materials and can be promptly extended to explore the effects of external perturbations such as strain, carrier doping, or substrate-induced modifications.
